# Evolution of Ebola Virus Disease from Exotic Infection to Global Health Priority, Liberia, Mid-2014

**DOI:** 10.3201/eid2104.141940

**Published:** 2015-04

**Authors:** M. Allison Arwady, Luke Bawo, Jennifer C. Hunter, Moses Massaquoi, Almea Matanock, Bernice Dahn, Patrick Ayscue, Tolbert Nyenswah, Joseph D. Forrester, Lisa E. Hensley, Benjamin Monroe, Randal J. Schoepp, Tai-Ho Chen, Kurt E. Schaecher, Thomas George, Edward Rouse, Ilana J. Schafer, Satish K. Pillai, Kevin M. De Cock

**Affiliations:** Centers for Disease Control and Prevention, Atlanta, Georgia, USA (M.A. Arwady, J.C. Hunter, A. Matanock, P. Ayscue, J.D. Forrester, B. Monroe, T.-H. Chen, T. George, E. Rouse, I.J. Schafer, S.K. Pillai, K.M. De Cock);; Ministry of Health and Social Welfare, Monrovia, Liberia (L. Bawo, M. Massaquoi, B. Dahn, T. Nyenswah);; National Institutes of Health, Bethesda, Maryland, USA (L.E. Hensley);; US Army Medical Research Institute of Infectious Diseases, Frederick, Maryland, USA (R.J. Schoepp, K.E. Schaecher)

**Keywords:** Liberia, West Africa, hemorrhagic fever, Ebola, disease outbreaks, epidemiology, public health, Filoviridae, Ebolavirus, viruses, zoonoses, expedited, Liberian Ministry of Health and Social Welfare, Ebola virus disease

## Abstract

Over the span of a few weeks during July and August 2014, events in West Africa changed perceptions of Ebola virus disease (EVD) from an exotic tropical disease to a priority for global health security. We describe observations during that time of a field team from the Centers for Disease Control and Prevention and personnel of the Liberian Ministry of Health and Social Welfare. We outline the early epidemiology of EVD within Liberia, including the practical limitations on surveillance and the effect on the country’s health care system, such as infections among health care workers. During this time, priorities included strengthening EVD surveillance; establishing safe settings for EVD patient care (and considering alternative isolation and care models when Ebola Treatment Units were overwhelmed); improving infection control practices; establishing an incident management system; and working with Liberian airport authorities to implement EVD screening of departing passengers.

## Abstract 2

As the disease spread, the scale of the epidemic required a multi-faceted public health response.

“Reviewing that first phase in the light of subsequent events, our townsfolk realized that they had never dreamed it possible that our little town should be chosen out for the scene of such grotesque happenings as the wholesale death of rats in broad daylight or the decease of concierges through exotic maladies.”

—Albert Camus, The Plague (1948)

The Ebola virus disease (EVD) epidemic in West Africa is recognized as the largest in history; more cases reported than in all previous EVD outbreaks combined (*1*). However, until the summer of 2014, the epidemic had not captured the world’s attention.

The EVD epidemic began in Guinea in late 2013. In neighboring Liberia, EVD was first diagnosed in a patient in mid-March 2014. A team of epidemiologists from the US Centers for Disease Control and Prevention (CDC) began working with the Liberian Ministry of Health and Social Welfare (MOHSW) in early April. By April 9, a total of 12 EVD cases (6 laboratory-confirmed) had been identified in Liberia, but no additional cases were reported in April or during most of May, and it appeared that the outbreak had been contained locally. However, on May 25, a patient who had traveled from Sierra Leone died of suspected EVD in Lofa County in northern Liberia. Within days, additional EVD cases were reported in Lofa County and in Monrovia, the capital city of Liberia. The MOHSW initiated investigations into what was considered a second epidemic wave of EVD.

## Background

**Figure 1:**
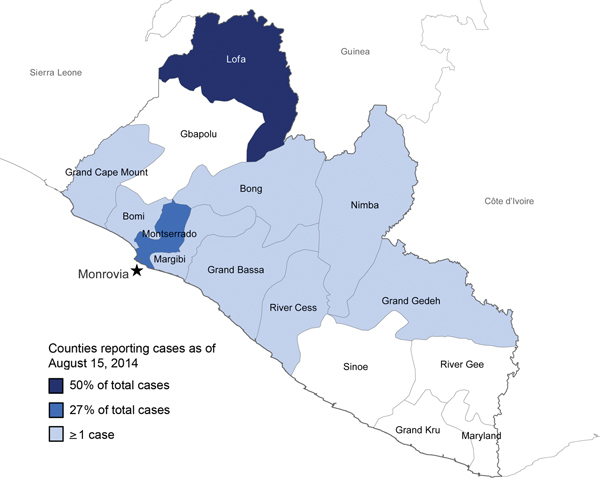
Counties in Liberia reporting Ebola virus disease cases as of August 15, 2014. Star indicates the capital city, Monrovia.

Liberia is a West African country of ≈4 million that is bordered by Guinea, Sierra Leone, and Côte d’Ivoire (Figure 1). Administratively, the country is divided into 15 counties; Monrovia makes up most of Montserrado County and accounts for ≈25% of the country’s population. Liberia ranks 175th of 187 countries in human development (*2*) and, reporting a gross domestic product per capita of US$454 per year, is 181st of 185 countries surveyed (*3*). During 1989–2003, civil war destroyed much of the country’s infrastructure and left a generation without education: the adult literacy rate is 43% (*4*). Before the EVD epidemic, the country had <200 physicians (*5*).

At the request of the government of Liberia, CDC sent a second team to Liberia in mid-July with expertise in epidemiology, logistics, border health measures, and health education. Team members worked in the 6 counties in Liberia that had reported EVD cases at that point, as well as in 4 counties that had not yet been affected, to assess the extent of the outbreak, preparedness, and general needs. CDC staff worked with county health teams and facilities, investigated clusters of EVD cases, and worked with the MOHSW to strengthen national surveillance for EVD and to support establishment of an incident management system. Team members also worked with Liberian airport authorities to implement Ebola virus (EBOV) screening of departing passengers. We describe the team’s field observations from mid-July to mid-August, 2014.

## Surveillance, Data, and Logistical Challenges

“… so that he should not be one of those who hold their peace but should bear witness in favor of those plague-stricken people…”

—Albert Camus, The Plague

The MOHSW compiled epidemic data, primarily received by telephone from county surveillance officers and supplemented by paper case report forms, into daily national situation reports. However, because of severely limited human and material resources, local health teams often prioritized case management, safe burial, and community education efforts over comprehensive data collection and reporting; therefore, official case counts underestimated disease incidence. In an extreme example, the daily national situation report listed 4 EVD cases from 1 county at a time when the county’s health team was aware of >80 cases (M.A. Arwady, unpub. data). This county’s surveillance officer had died; the public hospital had ceased operations after a large cluster of cases among its health care workers (HCWs); and the capabilities for both response and reporting had been overwhelmed. Improving EVD national surveillance was therefore a priority during July and August.

The MOHSW established public telephone hotlines, designed to aid in case finding by capturing information about ill or deceased persons with suspected EVD in the community. However, because <10 clinical and burial teams were available to dispatch to callers, assistance was often delayed by days, and the hotlines were overwhelmed by hundreds of calls daily from frustrated citizens.

Each day brought new local crises: counties exhausted their supplies of basic materials (gloves, body bags, and bleach); county employees evaluated threats from fearful and angry community members, searched for vehicles to carry bodies and transport patients, and worked to build isolation facilities and secure pay for health workers. In some settings, patients’ families or entire communities refused to allow health care workers access, fearing the workers would bring illness.

The scale of the epidemic made the comprehensive data collection and data entry processes used in earlier EVD outbreaks impractical. In some instances, patient data could not be obtained because persons with EVD did not seek clinical care or were turned away from overburdened facilities. Occasionally, whole families died before public health workers could interview them. Particularly in rural areas, problems with copying and transporting paper forms limited timeliness of reporting, and intermittent power supply and limited cell phone and internet service hindered connectivity.

**Figure 2:**
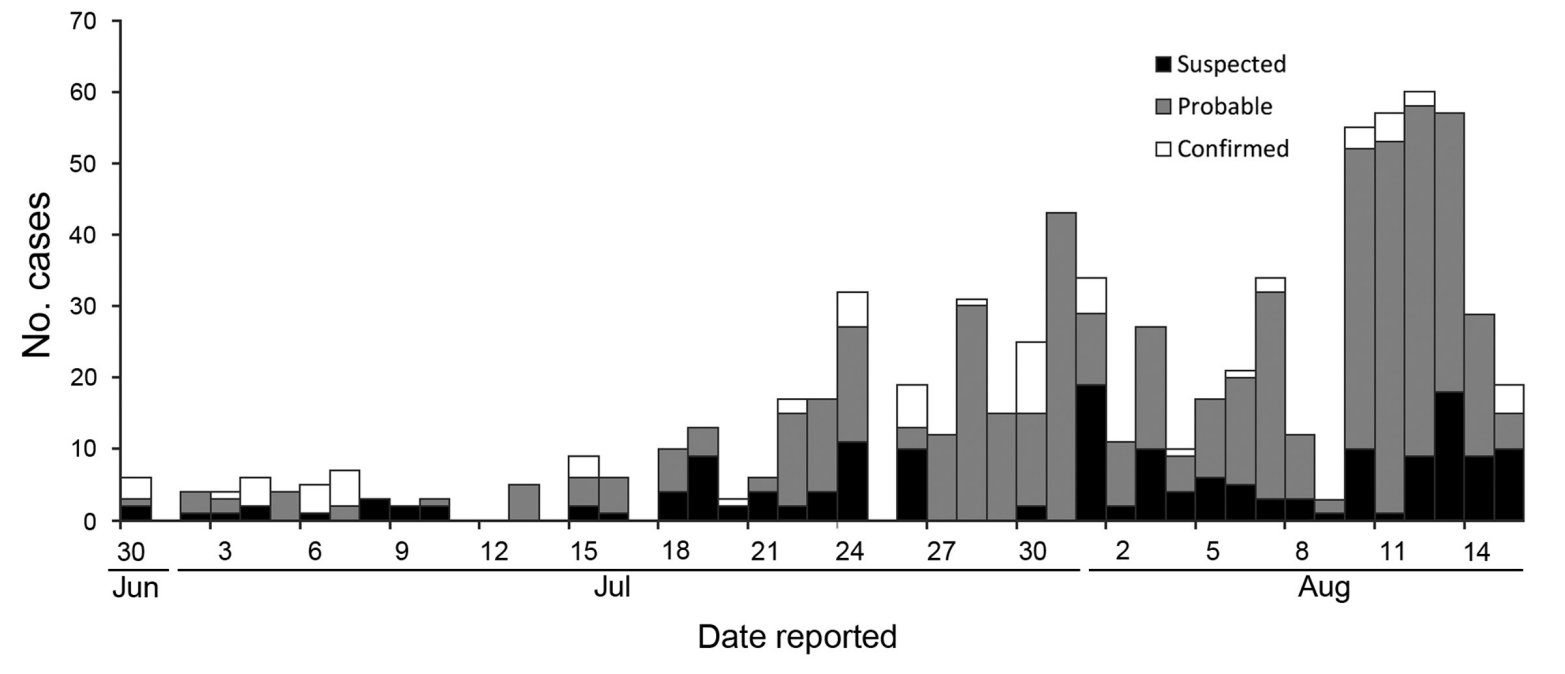
Reported Ebola virus disease cases by date, June 30–August 15, 2014, Liberia (n = 826).

**Figure 3:**
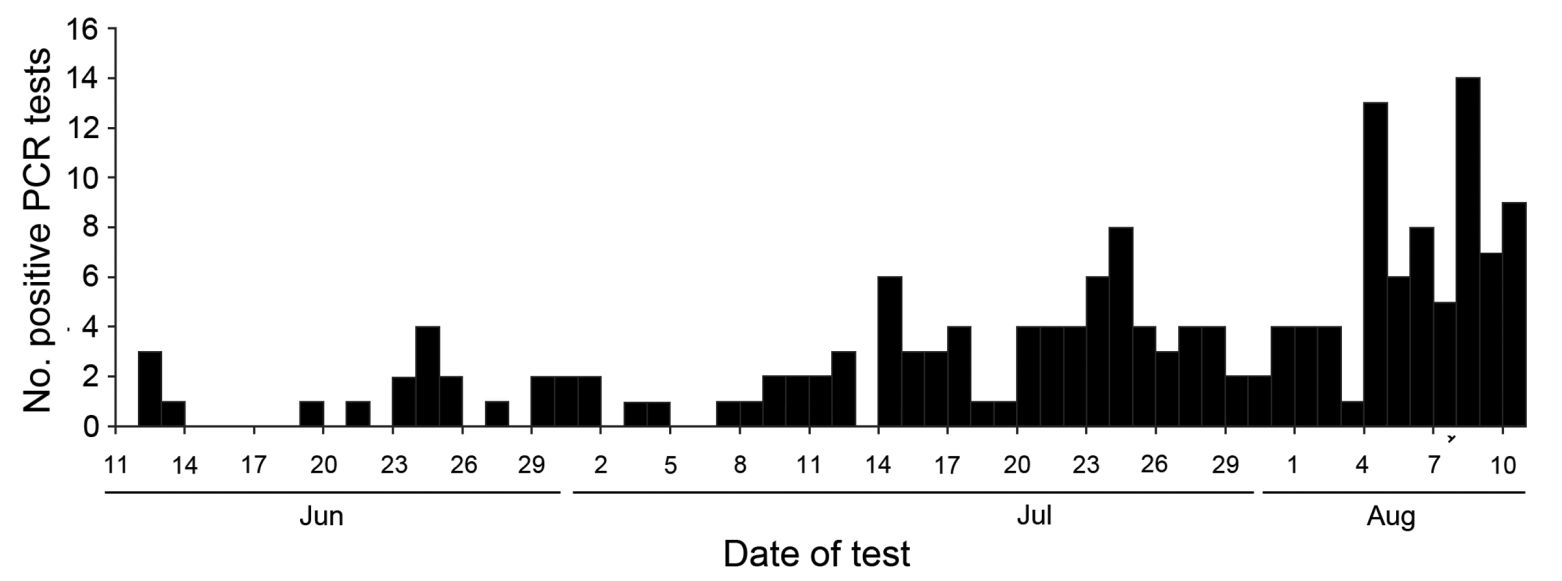
Positive PCR tests for Ebola virus infection, Liberia Institute for Biomedical Research, June 1–August 10, 2014 (n = 172).

Even with these known data limitations, the trajectory of the epidemic was clear. The number of counties reporting cases during July and August increased from 6 to 10. The MOHSW situation report dated July 15, 2014, described 173 EVD cases in detail (including suspect, probable, and laboratory-confirmed cases); over the next 30 days, a 4.8-fold increase in cases occurred, with a mean of 23 new cases and 12 deaths reported daily (range 3–60 new cases and 0–33 deaths per day). By August 15, 826 cases and 455 deaths (55.1% case-fatality rate) had been reported to the MOHSW (Figure 2). Of the reported cases, 23.5% were laboratory confirmed (Figure 3).

**Figure 4:**
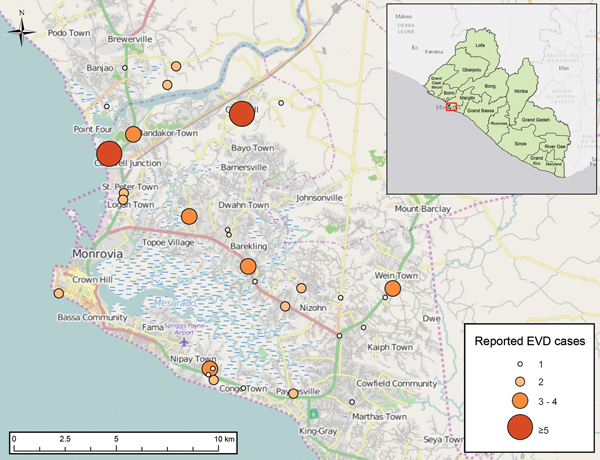
Reported Ebola virus disease cases, Montserrado County, Liberia, as of July 31, 2014.

The second wave of the EVD epidemic in Liberia started in semi-rural Lofa County in late May 2014, as the epidemic spread in the cross-border area of Guinea, Sierra Leone, and Liberia. As of the August 15 MOHSW situation report, 409 (50%) of Liberia’s 826 reported EVD cases were from Lofa County (Figure 1). EVD was likely introduced into Monrovia by infected persons traveling from Lofa County, Guinea, or Sierra Leone; Monrovia then became the second reservoir of infection in Liberia. By July 31, cases had been reported across much of the capital city (Figure 4). As of August 15, Montserrado County, which includes Monrovia, had reported 223 cases, accounting for 27% of total cases. Anecdotes suggested that many more cases were occurring in Monrovia than were being captured in official reports (M.A. Arwady, unpub. data). In the less-affected counties, infected travelers probably introduced localized outbreaks, and EVD was identified in health care settings in areas of Liberia where EVD had not been previously reported; EBOV infection was potentially propagated in these facilities. As of August 15, when approximately half of the country’s cases had been reported in Lofa County and ≈25% in Montserrado County, the 8 other affected counties (Bong, Bomi, Grand Bassa, Grand Cape Mount, Grand Geddeh, Margibi, Nimba, and River Cess) together accounted for 194 cases (23%), with a range of 1–87 cases per county. Five counties, predominantly in the southeast, had at that point reported no cases.

## EVD among Health Care Workers

“It could be only the record of what had had to be done, and what assuredly would have to be done… despite their personal afflictions, by all who, while unable to be saints but refusing to bow down to pestilences, strive their utmost to be healers.”

—Albert Camus, The Plague

By August 14, a total of 97 EVD cases had been reported in HCWs in Liberia, representing 12% of the 810 reported EVD cases nationwide. Rates varied by county: HCWs represented 4% of EVD cases in Lofa County, 17% in Montserrado County, and 20% in the other affected counties combined. Many of the earliest cases in newly affected counties were identified in HCWs, after infected travelers sought care in hospitals that had not previously seen patients with EVD and where HCWs provided care with minimal or no personal protective equipment (PPE). The CDC team investigated 10 clusters of EVD among HCWs in 4 counties. The number of HCWs per cluster ranged from 2 to 22, with a median of 5. Nurses and nurse’s aides accounted for 35% of infected HCWs; physicians and physician assistants accounted for 15%. Other occupations affected included laboratory workers, cleaners, hygienists, pharmacists, public health workers, and midwives.

In July and August, almost all health care facilities nationwide lacked standard infection control and preparedness procedures. Staff training was limited, and some at-risk workers such as cleaners had not received any training. PPE was generally not available in adequate quantities outside of the country’s 2 Ebola treatment units (ETUs), and chlorine disinfectant was in short supply. Almost all hospitals lacked appropriate isolation facilities for suspected case-patients, although by August, many hospitals were beginning to construct or refurbish temporary isolation units.

Many referral hospitals and smaller health facilities ceased operations in July and August because staff and non-EVD patients abandoned facilities in which EVD cases had occurred. In many areas, emergency care and basic outpatient services including prenatal care, HIV/AIDS services, and vaccinations were no longer available. The effects of cessation of these services on overall health outcomes, such as infant or maternal death rates, have not been formally assessed, but it is likely that rates of illness and death from non-EVD illnesses increased. Although the MOHSW emphasized the importance of re-opening facilities to provide essential services, HCWs voiced the need for enhanced infection control, adequate PPE and training, and, in some cases, financial compensation for their increased level of risk.

## Establishment of Liberia’s Incident Management System

“Everybody knows that pestilences have a way of recurring in the world; yet somehow we find it hard to believe in ones that crash down on our heads from a blue sky. There have been as many plagues as wars in history; yet always plagues and wars take people equally by surprise.”

—Albert Camus, The Plague

When the CDC team arrived in July 2014, the MOHSW was meeting daily with a group of in-country and international responders designated as the Ebola Task Force. The meetings were open, sometimes attracting nearly 100 persons, and were attended by technical staff involved in all aspects of the response, including staff from partner organizations such as the World Health Organization (WHO), Médecins Sans Frontières (MSF), and others (see complete list at the end of this article). Although the Task Force was intended to be national in oversight, meetings sometimes focused on local operational crises, such as a lack of vehicles for collecting bodies around Monrovia, and there was not a clear system for ensuring that agreed-upon actions had been implemented.

The MOHSW worked with the CDC team to establish an incident management system, which is a standardized tool for responding to emergencies under which personnel, resources, and logistic support are organized and managed according to specific objectives. The MOHSW appointed an incident manager, whose sole responsibility was to identify key objectives and lead the response, and a deputy incident manager to coordinate county activities. Task force meetings were reorganized to limit attendance to members of essential response committees that were empowered to make decisions and to include time for follow-up on outcomes of tasks assigned the day before. Key international partners, such as WHO, United Nations Mission in Liberia, CDC, and the United States Agency for International Development, also designated 1 representative each with decision-making capability. Documentation of daily assigned tasks and separation of national and county-level priorities improved organizational efficiency and effectiveness.

## EVD Treatment Units, Laboratory Capacity, and the Provision of Care

“As for the ‘specially equipped’ wards, he knew what they amounted to: two outbuildings from which the other patients had been hastily evacuated… The only hope was that the outbreak would die a natural death…”

—Albert Camus, The Plague

The standard approach to controlling EVD epidemics depends on active case finding and isolation of patients, with identification and careful monitoring of their contacts and immediate isolation of any contacts who develop symptoms. Isolation units serve to remove infectious persons from the community and to provide patients with supportive care. In previous EVD outbreaks, ETUs have been established and staffed by international health workers with specialized expertise from organizations such as MSF, supported by local employees.

In late July, only two 20-bed ETUs were operating in Liberia: 1 in Foya, northern Lofa County, and the other adjacent to Eternal Love Winning Africa Hospital, known as ELWA, in Monrovia, Montserrado County. A third ETU at John F. Kennedy (JFK) Hospital in Monrovia closed in mid-July after infections were diagnosed in staff members and had not reopened a month later. Both operational ETUs were directed by Samaritan’s Purse, an American missionary organization with a long history of providing health care in Liberia. Samaritan’s Purse staff had been trained by MSF, which continued to provide technical support in both ETUs. In late July, Samaritan’s Purse withdrew from Liberia after a cluster of EVD among staff members, and the ETUs then depended largely on MOHSW staff, with limited international support.

Increasing numbers of EVD cases overwhelmed the capacity of both ETUs, making them unable to accept patient transfers from other counties. On August 14, at the 20-bed ETU in Monrovia, >80 patients were on the premises, including ill patients lying on the grounds outside the facility waiting for a bed. Fifteen bodies were awaiting collection and burial. MOHSW Surveillance Unit data from mid-August indicated that only 25% of all reported EVD case-patients had been admitted to ETUs, but given underreporting of cases, the actual proportion of persons with EVD who reached an ETU is likely even lower. A high case-fatality rate in the Monrovia ETU, possibly the result of inadequate staffing in the face of an overwhelming caseload and concurrent delays in patients seeking care, suggested that under these operational constraints, the units were providing little clinical benefit. The basics of EBOV containment (i.e., isolation of cases and contact tracing) failed because of the large number of cases, insufficient number of isolation beds, and inability to track all contacts or isolate them if they became ill.

Laboratory capacity was also limited. Until late August, the Liberia Institute for Biomedical Research laboratory outside Monrovia was the only laboratory in the country performing EBOV testing. At the initial staffing levels, only 30–40 specimens could be tested per day; in addition, the laboratory was a full day’s drive from many outlying areas, and counties had few vehicles for transporting laboratory specimens or patients. Specimens from the ETU in Lofa County were sent across the border to Guéckédou, Guinea, for testing in the laboratory there, which was supported by the European Union. This arrangement was threatened in mid-August by an increase in the number of specimens requiring testing and by international border closures.

## Priority for Global Health Security

“…when the most pessimistic had fixed it at, say, six months; … a flash of foresight would suggest that, after all, there was no reason why the epidemic shouldn’t last more than six months; why not a year, or even more?”

—Albert Camus, The Plague

In the span of a few weeks in July and August, 5 sets of circumstances in West Africa changed perceptions of EVD from an exotic tropical disease to a priority for global health security. First, on July 20, a Liberian-American who had EVD traveled by air from Monrovia to Lagos, Nigeria. His arrival and subsequent care resulted in an EVD outbreak in Nigeria that resulted in 20 cases and required public health authorities to follow up on nearly 900 contacts to successfully contain the outbreak. After this exported EVD case, temperature screening and a health questionnaire for outbound passengers were instituted at Liberian airports. MOHSW, airport authorities, CDC, and private partners coordinated support to enhance safety and continuity of commercial air traffic into and out of Liberia as international attention increased.

Second, the late July diagnosis of EVD in 2 persons from the United States who were working in an ETU in Monrovia, their evacuation to the United States in early August, and their receipt of an investigational therapy aroused further international media interest. The situation highlighted the need for decisions concerning therapeutic and vaccine research during this epidemic and for defined policies by international organizations on evacuation of staff in the event of EBOV exposure or infection.

Third, local security concerns emerged in July and August. On July 23, for example, MOHSW employees and CDC team members had to evacuate the MOHSW after a relative of a person who died from EVD set the building on fire. In late July, a CDC team member and other international responders urgently crossed into Guinea from Lofa County after a burial team was attacked and its vehicle destroyed. On August 6, the president of Liberia declared a national state of emergency, and later in August, a nationwide curfew was established. On August 20, looting of an isolation facility in West Point, Monrovia, led to police gunfire and a death.

Fourth, responders recognized that adequate isolation facilities and a county-specific or community-specific containment approach were essential, and that these needs required additional resources and new approaches. With the resources available in July and August, medical relief, public health, and other organizations were unable to provide the trained personnel and specialized resources required to establish new ETUs in all places where they were needed, and other models of isolation and care had to be considered. Preliminary discussions were held with MOHSW, MSF, CDC, WHO, and other organizations about establishing lower level isolation units in community settings (e.g., schools) and the provision of home-based care for patients unable or unwilling to be evacuated.

Fifth, during this time period it became clear that, to have any chance of containment, the response would have to be increased by several orders of magnitude. By August, MSF had concluded that it could not provide the usual level of care given in other EVD outbreaks with the available resources and repeatedly stressed the inadequacy of the global response. On August 8, the Director-General of WHO declared that conditions for a public health emergency of international concern had been met (http://who.int/mediacentre/news/statements/2014/ebola-20140808/en/). Even as the global response increased, adverse epidemiologic trends seen in July and August worsened, and case counts through September increased exponentially. The sheer number of cases continued to outstrip efforts at active case finding and contact tracing, and ill persons continued to be turned away from hospitals and ETUs that had no beds. By September, EVD was widespread across the country, and cases doubled nationwide every 15–20 days.

## Discussion

The unprecedented scale of this epidemic has indirectly affected both the health system and daily life in Liberia. Beginning in July 2014, the government of Liberia closed all schools, canceled sporting and community recreation events, and restricted public gatherings to limit the spread of infection. Because hospitals with EVD cases closed or were abandoned and many health clinics were temporarily shut, many Liberians avoided health care settings out of fear of infection. The long-term effect on public health will become evident with time, although adverse effects must be expected, including deterioration of core public health services such as routine immunizations, tuberculosis programs, HIV/AIDS treatment, and maternal and child health services. As hospitals reopen for regular care, adequate patient triage, PPE, and HCW training remain essential. Enhanced infection control must continue to be a major component of the national response, and protecting HCWs must be of paramount importance. The vulnerability from lack of proper infection control practices and inadequate PPE in health care settings is a strong lesson for other African countries as they prepare for potential EVD introduction.

Thousands of people in West Africa continue to work tirelessly to fight this epidemic. In Liberia, the MOHSW has continued to adjust strategies and organizational structures as it leads the response. Other CDC teams replaced the July–August team in Liberia; the West African EVD response is already the largest international outbreak response in CDC’s history (*6*). However, in the same way that Liberia has isolated certain areas internally, the country itself faces isolation from the rest of the world. Fears around exposure to EBOV in social settings and lack of available medical care for conditions other than EVD prompted many expatriates and privileged Liberians to leave the country. Despite ongoing passenger screening programs, many airlines ceased flying into Monrovia, and some countries are refusing entry of travelers from Liberia. The economy has visibly contracted, prices of commodities and certain food items have increased, and national food security is an ongoing concern.

A “before and after” moment, before the West African EVD epidemic and after, has occurred in global health. The EVD epidemic in Liberia and other parts of West Africa reinforces the reality of global interconnectedness and common vulnerability from the weakest links in the chain, and remains a formidable challenge to the political and humanitarian solidarity of Africa and of the world. Continued support is required for efforts to implement and sustain effective border health measures to facilitate continued travel to the region, which is essential for the necessary flow of humanitarian aid.
